# Recognition of Human Oncogenic Viruses by Host Pattern-Recognition Receptors

**DOI:** 10.3389/fimmu.2014.00353

**Published:** 2014-07-22

**Authors:** Nelson C. Di Paolo

**Affiliations:** ^1^Lowance Center for Human Immunology, Division of Rheumatology, Departments of Pediatrics and Medicine, Emory University, Atlanta, GA, USA

**Keywords:** PRRs, oncogenic viruses, cancer, innate immunity, innate sensors

## Abstract

Human oncogenic viruses include Epstein–Barr virus, hepatitis B virus, hepatitis C virus, human papilloma virus, human T-cell lymphotropic virus, Kaposi’s associated sarcoma virus, and Merkel cell polyomavirus. It would be expected that during virus–host interaction, the immune system would recognize these pathogens and eliminate them. However, through evolution, these viruses have developed a number of strategies to avoid such an outcome and successfully establish chronic infections. The persistent nature of the infection caused by these viruses is associated with their oncogenic potential. In this article, we will review the latest information on the interaction between oncogenic viruses and the innate immune system of the host. In particular, we will summarize the available knowledge on the recognition by host pattern-recognition receptors of pathogen-associated molecular patterns present in the incoming viral particle or generated during the virus’ life cycle. We will also review the data on the recognition of cell-derived danger associated molecular patterns generated during the virus infection that may impact the outcome of the host–pathogen interaction and the development cancer.

## Introduction

Seven human viruses have been found so far to cause approximately 10–20% of human cancers worldwide (1). They include the herpesviruses, Epstein–Barr virus (EBV) and Kaposi’s associated sarcoma virus (KSHV), the hepatitis B (HBV) and hepatitis C (HCV) viruses, high-risk human papillomaviruses (HPV) (the most clinically important ones being types 16 and 18, but most probably a few others will be found to be relevant to cancer development as well in the future), the human T-cell lymphotropic virus-1 (HTLV-1), and the recently discovered Merkel cell polyomavirus (MCPyV) (1). The mechanisms by which these viruses cause cancer are diverse. They have prolonged latency periods, during which viral factors combine with other environmental factors in the setting of the genetic background of each particular host (2). However, it could be proposed that these viruses have no intention of generating disease in their hosts, as evidenced by the overall rate of disease/infected humans worldwide for each virus (Table 1). Although exact numbers are not available for every region in the world, the number of humans that suffer a disease associated with each oncogenic virus, as compared to the number of people infected with each virus is evidently low. It appears that during evolution these viruses have found a balance of “live and let live” with their host. Until very recently in history, humans were not living long enough to considerably suffer from the diseases attributed to these viruses (3). Today, however, human longevity is greatly extended, and although the burden of diseases associated with oncogenic viruses is still low in comparison with the number of infected people, the goal of medicine is, of course, to eradicate diseases. Understanding the interactions of these viruses with the host will certainly help to achieve this goal. Of particular importance is their interaction with the innate immune system, which functions to recognize non-self like microorganisms, and also plays a critical role in recognition of modified self that indicates damage or danger (4).

**Table 1:** **Oncoviruses induce cancer in only a fraction of infected humans**.

Virus	Family	Infected worldwide (estimated)	Percentage developing disease*	Reference
HBV	Hepadnaviridae	400 million	HCC: 340,000/year (1% approximately)	Busca and Kumar (5)
HCV	Flaviviridae	210 million; 80% persistent infection	HCC: 195,000/year (1% approximately)	Eksioglu et al. (6)
EBV	Herpesviridae	90% Human population (approximately 6.3 billion?)	Most people do not develop disease	Zauner and Nadal (7)
KSHV	Herpesviridae	Not ubiquitous, perhaps between 5 and 50% of the population	Varies	Areste and Blackbourn (8)
HPV	Papillomaviridae	50–80% Of sexually active adults; one or more HPV types during lifetime	Invasive cervical cancer: 500,000 cases/year	Sunthamala et al. (9)
HTLV-1	Retroviridae	10–20 million	2–3% ATL; 0.25–4% HAM/TSP	Oliere et al. (10)
MCPyV	Polyomaviridae	Reports vary, between 20 and 80% of population tested	MCC: 1600 cases year in USA	Bhatia et al. (11)

*Numbers are approximate, and may vary in different geographical regions.

*HCC, hepatocellular carcinoma; MCC, Merkel cell carcinoma. See other abbreviations in text*.

Germline-encoded pattern-recognition receptors (PRRs) recognize chemically distinct moieties in microorganisms or “pathogen-associated molecular patterns” (PAMPs) (12). PRRs can also recognize endogenous host molecules that in different ways signal danger (“damage” or “danger”-associated molecular patterns’ or “DAMPs”) (13, 14). It is noteworthy that the “D” in DAMPs is used interchangeably for “danger” or “damage.” However, “danger” would seem to be more appropriate, as there could be danger without damage, and it would be more in line with the original “danger” theory proposed by Matzinger several years ago (15).

There are two families of transmembrane PRRs, namely toll-like receptors (TLRs) (16) and C-type lectin receptors (CLRs) (17). They are positioned to scan the extracellular and endosomal spaces. The families of *cytoplasmic* PRRs include the retinoic acid-inducible gene (RIG)-I-like receptors (RLRs) (18) and the nucleotide-binding, oligomerization domain (NOD)-like receptors (NLRs) (19, 20), as well as a large number of DNA sensors that converge in the adaptor for cytosolic DNA sensing stimulator of interferon genes (STING). An excellent very comprehensive review on nucleic acid sensing was recently published (21). The double-stranded (ds)RNA-dependent protein kinase R (PKR) and the 2′,5′-oligoadenylate synthetases (OAS) are considered part of the cytoplasmic PRRs as well (22). Recently, a *nuclear* DNA sensor was identified, IFI-16, a PYHIN protein that, together with the cytoplasmic AIM-2 DNA sensor, was proposed to form a new family of innate DNA sensors (“AIM2-like receptors” or “ALRs”) (23). Importantly, some of the members of the NLR and ALR families form a molecular complex termed “inflammasomes,” molecular platforms that control the secretion of the pro-inflammatory cytokines interleukin-1b and -18 (14). Some of the members of the already mentioned families of receptors recognize DNA. However, there is a growing list of DNA sensors not belonging to these families, recognizing both pathogen’s DNA as well as modified or displaced self DNA (24). Finally, although it is not be in the scope of this review, it is relevant to mention here that there is a particular set of immune proteins called “intrinsic antiviral factors.” Unlike PRRs that function against viruses by triggering a cascade of antiviral signaling events, intrinsic antiviral factors directly block viruses at different points of their life cycle (25). Each of these families of proteins does work in concert in order to eradicate viruses. However, viruses have evolved a myriad of mechanisms to evade and subvert these host antiviral defenses in order to ensure their evolutionary survival (26).

**Figure 1:**
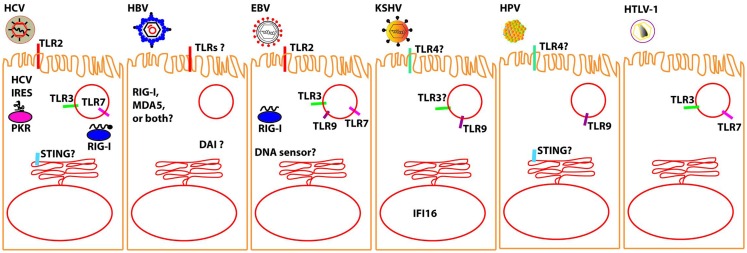
**Molecular sensors that have been proposed to detect oncolytic viruses (to the best of our knowledge, no sensor has been yet definitively shown for MCPyV)**. It should be noted that most of the interactions between the described oncolytic viruses and the proposed sensor awaits verification in relevant in vivo models. Question marks are intended to denote those sensors for which there is particularly conflicting data; please see specific sections in text for further details.

There is abundant information on the mechanisms by which the seven oncogenic viruses block the molecular pathways of the innate immune system at the level of intracellular adaptors, and the reader is referred to the several extensive published reviews in the specific sections below. However, much less is known on the recognition of these viruses by the sensors that physically interact with viral PAMPs. Here, we will focus on the latest findings on the growing list of innate immune sensors that have been implicated in sensing each known human oncogenic virus (Figure 1). We believe that by combining this information in one single review, parallelisms and differences between these very distinct viruses, which trigger the same human disease, i.e., cancer, may be revealed.

## Hepatitis C Virus

Hepatitis C virus is a single-stranded RNA virus, with an enveloped nucleocapsid of about 50 nm. It is transmitted via parenteral route, and there are millions of people infected with HCV worldwide, for which there is no available vaccine (27). During its evolution with the host, it has developed a number of mechanisms to avoid being eliminated by the innate immune system, establishing chronic infection of the liver. This chronic infection triggers injury to the liver, which is believed to be the basis for the development of liver cancer. HCV and its interaction with the adaptive and innate immune systems is a very active field of research, and many recent review articles have exhaustively discussed these topics (6, 27–32). However, the sensing of the virus and the innate pathways activated during the first days of infection in humans remain largely unknown (33). Understanding of these steps is critical, as they are likely to set the stage for the ultimate outcome of the infection.

### Cellular membrane and endosome sensing

TLR2 has been proposed to sense HCV proteins at the cell surface (30). TLR3 has been shown to be relevant for the activation of the transcription factors IRF-3 and NF-κB in response to HCV–RNA (28). TLR7 was also shown to be relevant in HCV sensing (34), and the proposed mechanism suggested the existence of a cell–cell RNA transfer process where HCV-infected cells activated plasmacytoid dendritic cells (pDCs) in *trans*. This was shown to be the case as well by Dreux et al., who reported the transfer of HCV–RNA containing exosomes from infected cells to pDCs (35).

### Intracellular sensing

Hepatitis C virus recognition in the cytosol is mediated by the host RNA-dependent PKR, which identifies an internal ribosome entry site (IRES) in HCV genome. In contrast, the virus’ 3′ poly-U/UC sequence, short dsRNA regions, and 5′ triphosphate of the uncapped HCV–RNA are recognized by RIG-I [reviewed in detail by Horner (28); Horner and Gale (29)]. A detailed analysis of the HCV–RNA that activates RIG-I was described by Schnell et al. (36), who reported a 34-nt poly-uridine “core” of the 5′-ppp poly-U/UC sequence as a critical structure for RIG-I activation. Recently, a new mechanism by which HCV controls interferon (IFN) induction was described, where RIG-I is ubiquitinated through the di-ubiquitin-like protein ISG15, one of the early interferon responsive genes (ISGs) (37). Other investigators, however, propose a different mechanism of RIG-I activation, where Riplet-mediated K63-linked polyubiquitination releases RIG-I RD autorepression, allowing the access of downstream signaling factors to the RIG-I protein (38). These differences in the proposed models of RIG-I activation may be due to the use of different cell types and experimental conditions. More recent data also suggest that the STING may be relevant for HCV recognition (39, 40). The mechanism these investigators propose implicates direct interaction of HCV NS4B with STING, blocking IFN beta production downstream of both STING and RIG-I. Finally, although human biopsies provide limited opportunities for mechanistic studies, they are critical since they allow a snapshot view of the tissue that is infected in the actual host. Consistent with this concept, Mozer-Lisewska et al. reported that in liver from patients with chronic hepatitis C infection, the expression of TLR1, 2, 4, NALP, and RIG-I helicase was markedly increased, suggesting that these PRRs may be important for the pathogenesis of chronic viral hepatitis by HCV in humans (41).

## Hepatitis B Virus

Hepatitis B virus genome consists of partial dsDNA, its nucleocapside is enveloped, and is transmitted via the parenteral route; although there is a vaccine available, millions of people are infected (27). The major challenge for mechanistic analysis of HBV interaction with the innate immune system is the lack of a suitable animal model. Woodchuck infected with the woodchuck hepatitis virus (WHV) (42) is an accepted study model, but available immunological tools are limited. Researchers use transfected cells or mice hydrodynamically injected with HBV replicative plasmids, but they cannot faithfully recapitulate the *in vivo* infection process. Even with these caveats in mind, the field is advancing toward an understanding of the interaction between HBV and the human innate immune system. Until recently, it was believed that the virus was just a stealth pathogen that could not be detected by PRRs (43–45). However, it is becoming clear that HBV just have a number of very efficient strategies to block innate immunity, and they were recently reviewed in Ref. (5, 46). Indirect data seem to support the fact that PRR sensing of HBV is important for HVB pathogenesis. For example, Guo et al. showed that transfection in cells with the plasmids expressing adaptors for PRRs signaling pathways (myeloid differentiation primary response gene 88, or MyD88), TIR-domain-containing adaptor-inducing beta interferon (TRIF), or the RIG-I/MDA5 adaptor, interferon promoter stimulator 1 (IPS-1), reduced HBV DNA and RNA levels (47). However, it is difficult to conclude that the data obtained in this *in vitro* system correlates with the behavior of the virus in naturally infected hosts.

### Cellular membrane and endosome sensing

Using the HBV/WHV model, Zhang et al. described that addition of TLR2 ligands activate NF-κB, PI3K/Akt, and different arms of the MAPK signaling pathways to induce pro-inflammatory cytokines, leading to the reduction of WHV replication and gene expression in HepG2.2.15 cells and primary woodchuck hepatocytes (48). However, in a previous study using an HBV transgenic mice model, a single intravenous injection of exogenous ligands specific for TLR2, TLR3, TLR4, TLR5, TLR7, and TLR9 showed that all of the ligands except for TLR2 inhibited HBV replication in the liver non-cytopathically in an alpha/beta IFN-dependent manner (49). Differences in these results could easily be attributed to the different model systems used, and warrant further investigation. In a more relevant study model, i.e., the chimpanzee, Lanford et al. showed that the small molecule GS-9620, which activates TLR7 signaling in immune cells, provided long-term suppression of serum and liver HBV DNA (50). Based on these and other results, TLR ligands are being developed as drugs for the treatment of chronic viral infections, including HBV (51).

### Intracellular sensing

RIG-I and MDA5 are important PRRs responsible for recognition of viral RNAs produced during viral infection, and represent targets for immunosuppression during HBV infection. Lu and Liao demonstrated that in human Huh7 cells transfected and in the livers of mice hydrodynamically injected with HBV replicative plasmids, the expression of MDA5, but not RIG-I, was increased, and it was the critical protein for HBV detection (52). It is interesting that mice heterozygous for MDA5 also had an increase in HBV replication, indicating the existence of a possible threshold in MDA5 expression level necessary for its function as a HBV sensor. In another study, Zhao et al. proposed that RIG-I, and not MDA5, is the protein involved in HBV sensing (53). Although it is not clear as yet which specific sensor is involved, viral RNA sensing in the cytoplasm is clearly occurring during HBV infection. Studies using hepatocytes (54), 293 cells (55), or the cytoplasmic fraction of HBx transgenic mouse livers (56) showed that hepatitis B virus X (HBX) protein interacts with MAVS (also called IPS-1, a critical molecule in RNA signaling pathways) (57), and prevents the induction of IFN genes. DNA sensing mechanisms are also likely to be relevant, since in the cell line Huh7, Chen et al. showed that DAI can inhibit HBV replication, where the inhibitory effect was associated with activation of NF-κB, and was independent of IRF-3 or cytokines (58).

In summary, it is clear that many more studies identifying new mechanisms of HBV detection by the innate immune system are likely to follow. The true challenge will be to reconcile those *in vitro* identified pathways with the mechanisms of HBV control in more relevant infectious models, i.e., the chimpanzee, and translate this knowledge into human settings.

## Herpesviruses: Epstein–Barr Virus and Kaposi’s Associated Sarcoma Virus

There is a significant body of data demonstrating that herpesviruses can be sensed by the innate immune system at the cellular membrane, in the endosomes, and in the cytosol. Furthermore, recent studies showed that herpesviruses can also be sensed in the nuclei. A recent comprehensive review on herpesviridae was published by Paludan and Bowie (24). EBV and KSHV are the two members of this virus family that have been identified as having growth transforming potential, and therefore, we focus on these here.

## Epstein–Barr Virus

Epstein–Barr virus was discovered approximately 50 years ago. It is an enveloped virus with a dsDNA genome, for which there is extensive knowledge about its biology (59). The innate immune recognition of EBV was also reviewed in detail (60, 61).

### Cellular membrane and endosome sensing

Epstein–Barr virus can be sensed by TLR2 in certain cells; however, the exact virion component being sensed is still unclear (62). Ariza et al. proposed that deoxyuridine triphosphate nucleotidohydrolase (dUTPase), a non-structural protein encoded by EBV, is sensed by TLR2 and initiates a MyD-88 dependent response (63). This group further extended their results to demonstrate that the protein was secreted in exosomes inducing NF-κB activation and cytokine secretion in primary DCs and peripheral blood mononuclear cells (PBMCs) (64). However, these results should be interpreted with caution given that the studies were done using an *in vitro* experimental system. EBV produces non-coding RNAs or “Epstein–Barr virus-encoded small RNA” (“EBER”). TLR3 is a sensor of viral dsRNA. Very interestingly, it was discovered that a substantial amount of EBER was released from EBV-infected cells in exosomes that stimulated DCs to produce type-I IFN. Most importantly, they found EBER in sera from patients with EBV-related diseases, suggesting that EBER could be responsible for immune activation by EBV, inducing type I IFN and proinflammatory cytokines (65). These results were further discussed by the same group (66). TLR7 has not been proposed as a direct sensor for EBV. However, Valente et al. reported that the aberrant activation of TLR7 in EBV-infected cells might induce the expression of the EBV-protein LMP1 (67). As LMP1 is known to prime cells to express IFN, and both TLR7 and IFNs are believe to be involved in the development of systemic lupus erythematosus (SLE, or simply “lupus”), the association of EBV infection and autoimmunity clearly warrants further investigation.

Interestingly, Severa et al. showed that EBV can activate pDCs through TLR9 and TLR7, in combination with functional autophagic machinery (68). However, these pDCs were not able to mature and induced an inefficient T-cell response, suggesting a new virus escape mechanism potentially related to EBV induced diseases. Another important finding reported by van Gent et al. showed that EBV encoded deubiquitinase, BPLF1, interferes with NF-κB activation mediated by TLR signaling (69). TLR9 can initiate a response by detecting EBV DNA in the endosomes. However, Fathallah et al. showed that EBV infection of human primary B cells results in the strong inhibition of TLR9 transcription by the EBV oncoprotein latent membrane protein 1 (LMP1) (70). The role of TLR9 in EBV infection has been exhaustively reviewed in Ref. (7).

### Intracellular sensing

In the cytosol, EBV EBERs are recognized by RIG-I (62). Moreover, RIG-I has been proposed to indirectly sense EBV DNA by recognizing the 5′-triphosphate transcribed by the host RNA polymerase III (71). However, there are conflicting results that need to be resolved by further experimentation to clarify the role of RNApol-III in EBV sensing mechanism (62). There are numerous DNA sensors in the cytosol, and although some of them have been shown to recognize other herpesviruses (62), the relevance of cytosolic DNA sensors to EBV remains unclear.

## Kaposi’s Associated Sarcoma Virus

This virus, formally classified as human herpesvirus 8 (HHV-8), is associated with Kaposi’s sarcoma (KS), among other pathologies (72). It is a big enveloped virus with a dsDNA genome (73). Employing many proteins and micro-RNAs, KSHV modulates the innate and adaptive immune system of the host at multiple levels. A number of excellent reviews on these topics have been recently published (8, 73–75).

### Cellular membrane and endosome sensing

Only recently, researchers have started investigating the role of TLR-mediated sensing of KSHV. Although a direct interaction of KSHV with a TLR has not been reported, the virus downregulates the expression of TLR4 soon after infection in endothelial cells (76). West and Damania, however, showed that in monocytes TLR3 expression is upregulated after KSHV infection (77). Gregory et al. showed that agonists specific for TLR7/8 reactivated latent KSHV and induced viral lytic gene transcription and replication (78). Moreover, the same was accomplished by secondary infection with vesicular stomatitis virus (VSV), which also activates those same TLRs. More recently, pDCs were shown to respond to KSHV in TLR9-dependent manner (79). Finally, it has been shown that stimulation of the TLR3–TRIF axis increases the expression of the KSHV protein RTA (replication and transcription activator), only for RTA to degrade TRIF in order to block the innate immune response (80, 81). Collectively, although these results do not demonstrate a direct interaction between KSHV and TLRs, they clearly indicate that there is a physiologically relevant interplay between them.

### Intracellular sensing

The field of intracellular sensing of KSHV has recently seen a number of very exciting discoveries. Gregory et al. reported that KSHV Orf63 blocks NLRP1-dependent innate immune responses, including caspase-1 activation and processing of interleukin-1 beta (IL-1beta) and IL-18, and significantly reduces NLRP1-dependent cell death (82). Moreover, the inhibition of Orf63 expression resulted in increased expression of IL-1beta during the KSHV infection that could have an effect on KSHV induced pathologies. In a new development in the field of innate immune sensing, Unterholzner et al. reported that IFI-16 acts as a nuclear sensor for HSV-1 (23). Based on their findings, they proposed the existence of a new family of “AIM-2 like receptors” or ALRs. In the same line of research, Kerur et al. found that the same protein is responsible for KSHV sensing through an IFI-16/ASC inflammasome assembled in the nuclei (83). They reported that caspase-1 activation is IFI-16/ASC inflammasome dependent, and it leads to IL-1b secretion. Moreover, the same group proposed that latent KSHV genome is continuously sensed in the nuclei through IFI-16 sensing mechanism (84). Further studies will be needed to shed light on the biological significance of these very exciting findings. Finally, West et al. suggested a role for MAVS and RIG-I dependent signaling mechanisms during KSHV infection (85). Therefore, all of the families of cytosolic sensors have been implicated in the recognition of KSHV. These results clearly indicate that KSHV has a complex interaction with host innate immunity by activating several PRRs. It is conceivable that activation of this network of innate immune receptors is a necessary step in the virus pathogenesis to establish lifelong persistence of the virus infection.

## Human Papillomaviruses

The HPV family encompasses a large number of stable dsDNA viruses (86). Infections with high-risk HPVs are causally associated with the development of anogenital cancers (87). It has been proposed that HPVs evade the innate immune response of the host cells by deregulating immunomodulatory factors such as cytokines and chemokines, thereby creating a microenvironment that favors malignancy (88). The combination of knowledge from the fields of basic HPV virology and vaccinology was the driving force for the successful development of clinically effective vaccines against HPV (89). However, the developed vaccines are prophylactic, not therapeutic, and cover only a subset of HVP types. It is certainly clear that improving our understanding of the interaction of HPV with the innate immune system will improve the probability of success in developing better treatments. Similar to all other viruses described in this review, experimental systems that would be informative about HPV pathogenesis in humans are very limited. The vast majority of studies were performed using virus like particles (VLPs). This approach, and the differences between laboratories in their techniques for virus particles preparation, is partially responsible for the incomplete understanding of HPV biology. For example, the exact mechanism of virus entry into the cell remains incompletely defined (90). Along the same lines, the full spectrum of PRRs relevant to HPV recognition by the cell is yet to be determined.

### Cellular membrane and endosome sensing

The current understanding of the interaction between HPV and PRRs is mostly based on studies aimed to potentiate immunological responses to HPV vaccines by modulating innate immunity. Therefore, research in the field has focused primarily on the role of TLRs. To date there are no publications on the involvement of cytosolic or nuclear sensors in HPV recognition. There is currently no evidence that any cellular PRRs interact with HPV directly [reviewed in Ref. (88)]. A comprehensive review on the role of TLRs in HPV infection has been recently published by Zhou et al. (91). Although TLR4 was suggested to bind HPV L1 directly, these studies were performed using VLPs, and although TLR9 may recognize HPV DNA in the endosomes, it is not clear whether the HPV DNA is exposed in the endosome during natural viral infections (91). More recently, it was described that an HPV16 transcriptional repressor complex associates with the TLR9 promoter, suggesting that blocking this TLR-mediated sensing pathway may be of significance for the virus pathogenesis (92). Collectively, these data indicate that although direct interaction between HPV and PRRs is yet to be shown, the virus does interfere with innate pathogen recognition machinery. In this regard, several recent publications describing how HPV may control cellular responses initiated by PRRs pathways should be mentioned. IL-1beta is a critical cytokine that mediates inflammation and is important for both innate and adaptive immunity. Using immortalized keratinocytes, it was shown that the high-risk HPV16 E6 oncoprotein can abrogate IL-1beta processing and secretion independently of the NALP3 inflammasome (93). The authors further demonstrated that pro-IL-1beta is degraded by a novel proteasome-dependent mechanism via the ubiquitin ligase E6-AP and p53. Moreover, in a panel of HPV-positive tissue samples, the authors found correlation between reduced amounts of IL-1beta and the stage of cellular progression toward cervical cancer (93). HPV was also shown to interfere with innate immune signaling pathways through virus-dependent upregulation of an intrinsic ubiquitin ligase, ubiquitin carboxyl-terminal hydrolase L1 (UCHL1). Upregulation of UCHL1 inhibited TRAF-3 dependent phosphorylation of interferon regulatory factor-3 (IRF-3), and the activation of NF-κB (94). However, the role of this ubiquitin ligase *in vivo* remains unclear as these studies were performed in HPV infected keratinocytes. Using an *in vitro* approach, Sunthamala et al. found that HPV E2 protein interferes with innate immune signaling pathways by downregulating STING and IFN-κ (9). Importantly, they also demonstrated in clinical specimens that STING and IFN-κ are downregulated in HPV low grade lesions when compared to normal tissues. Conceptually and mechanistically interesting findings were made by Kumar et al., who showed that Langerhans cells from cervical tumors lack TLR9 expression and are functionally anergic to TLR7, TLR8, and TLR9 ligands (95). These data suggest that apart from directly interacting with cellular PRRs, HPV may interfere with innate signaling pathways in neighboring cells in an indirect paracrine manner leading to PRRs signaling inhibition.

### Human T-cell lymphotropic virus

Human T-cell lymphotropic virus-1 belongs to the retroviridae family and is an enveloped, round shaped particle with a single-stranded RNA genome (96). The diseases that induce are diverse and this diversity in clinical manifestations in response to HTLV-1 is likely associated with genetic heterogeneity of the host. The pathologies induced by this virus include the aggressive, fatal T-cell malignancy adult T-cell leukemia (ATL) and a chronic, progressive neurologic disorder called HTLV-1-associated myelopathy/tropical spastic paraparesis (HAM/TSP), among others. Unfortunately, the molecular mechanisms underlying the diversity in host responses to HTLV-1 remain unclear (96). The studies of host defense against HTLV-1 have largely focused on understanding the very strong CTL response against the virus. It is puzzling how the virus can establish a persistent infection in the face of such a response. One of the potential mechanisms to escape from the CTL response is the capacity of the virus to downregulate the expression of all but one viral protein (HBZ), thus directly reducing the immunogenicity of the infected cells (97). This capacity of the virus also makes its detection by the innate immune system very challenging. Several reviews have recently summarized the advances in the field of HTLV-1 interactions with the innate immune system (10, 97–99).

The fact that there is not an adequate animal model to study the virus interaction with innate immunity makes advancing in the field very challenging. Rabbits and monkeys models can be used; however, the available immunological tools are scarce. For HTLV-1, mice represent a very poor animal model. Finally, in contrast to the availability of human cervix samples for studies of HPV pathogenesis, access to central nervous system tissue of HTLV-1 infected individuals is not available (100, 101). Therefore, it is not surprising that as of yet there is no evidence of direct recognition of HTLV-1 by PRRs. Furthermore, the role of innate immunity in HTLV-1-associated diseases is not clear (99). Only recently, the induction of an innate immune response to HTLV-1 (102) was reported for the first time. The authors found that cell-free HTLV-1 stimulates pDCs to produce massive amounts of type-I IFN. The proposed mechanism of type-1 IFN induction was the degradation of the viral particles in the endosomal compartments, and consequent exposure of the ssRNA to TLR7. This model was supported by the indirect observations that an endosomal acidification inhibitor and a TLR7 specific blocker drastically inhibited pDC response to HTLV-1 measured by type-1 IFN production. Progress in understanding the innate immune responses to HTLV-1 may come from the use of humanized mouse models (100). For example, reconstitution of mice with WT or TLR7 deficient human cells may reveal the contribution of the TLR7 innate immune signaling pathway to recognition of HTLV-1.

## Merkel Cell Polyomavirus

Merkel cell carcinoma (MCC) is a highly aggressive non-melanoma skin cancer arising from epidermal mechanoreceptor Merkel cells. In 2008, a novel human polyomavirus, MCPyV, was identified and is now implicated in MCC pathogenesis. Polyomaviruses are small, non-enveloped dsDNA viruses [for a detailed review on polyomaviruses and MCPyV in particular see Ref. (11, 46, 103, 104)]. Although little is known about this newly identified virus, it is plausible that, as with other oncogenic viruses, MCPyV has an array of mechanisms to block the innate immune responses. There is limited information on the innate immune recognition of this virus, as the field is in its infancy. It was reported that MCPyV large T antigen (LT) expression downregulates TLR9 expression in epithelial and MCC-derived cells (105), but nothing is known regarding the direct recognition of the virus by PRRs. More data are clearly needed on the interaction of this virus with the innate immune system.

## Conclusion

Over evolutionary times, the battle between the oncogenic viruses and their hosts has arrived at a balance that ensures the survival of both organisms. However, with the current advances in vaccinology and drug development, it is plausible to imagine that we are potentially getting closer to limiting the impact these seven viruses have on the population of the world. Although a complete understanding of all of the complexity of interactions with the native host for all of the oncogenic viruses discussed in this review is still lacking, it is clear that the innate immune system is able to recognize their presence through a network of sensors. Undoubtedly, the understanding of virus interactions with the innate immune system will aid in the development of effective treatments against these pathogens. More research is clearly warranted to devise effective approaches to harness the tools of the innate immune system for elimination of these viral pathogens without negatively affecting their hosts.

## Conflict of Interest Statement

The author declares that the research was conducted in the absence of any commercial or financial relationships that could be construed as a potential conflict of interest.
